# The Skeletal Phenotype of Chondroadherin Deficient Mice

**DOI:** 10.1371/journal.pone.0063080

**Published:** 2013-06-03

**Authors:** Lovisa Hessle, Gunhild A. Stordalen, Christina Wenglén, Christiane Petzold, Elizabeth K. Tanner, Sverre-Henning Brorson, Espen S. Baekkevold, Patrik Önnerfjord, Finn P. Reinholt, Dick Heinegård

**Affiliations:** 1 Sections of Molecular Skeletal Biology and Rheumatology, Department of Clinical Sciences Lund, Lund University, Lund, Sweden; 2 Department of Pathology, University of Oslo, and Oslo University Hospital, Rikshospitalet, Oslo, Norway; 3 Faculty of Odontology, University of Oslo, Oslo, Norway; 4 School of Engineering, University of Glasgow, Glasgow, United Kingdom; 5 Section of Orthopaedics, Department of Clinical Sciences Lund, Lund University, Lund, Sweden; University of Patras, Greece

## Abstract

Chondroadherin, a leucine rich repeat extracellular matrix protein with functions in cell to matrix interactions, binds cells via their α2β1 integrin as well as via cell surface proteoglycans, providing for different sets of signals to the cell. Additionally, the protein acts as an anchor to the matrix by binding tightly to collagens type I and II as well as type VI. We generated mice with inactivated chondroadherin gene to provide integrated studies of the role of the protein. The null mice presented distinct phenotypes with affected cartilage as well as bone. At 3–6 weeks of age the epiphyseal growth plate was widened most pronounced in the proliferative zone. The proteome of the femoral head articular cartilage at 4 months of age showed some distinct differences, with increased deposition of cartilage intermediate layer protein 1 and fibronectin in the chondroadherin deficient mice, more pronounced in the female. Other proteins show decreased levels in the deficient mice, particularly pronounced for matrilin-1, thrombospondin-1 and notably the members of the α1-antitrypsin family of proteinase inhibitors as well as for a member of the bone morphogenetic protein growth factor family. Thus, cartilage homeostasis is distinctly altered. The bone phenotype was expressed in several ways. The number of bone sialoprotein mRNA expressing cells in the proximal tibial metaphysic was decreased and the osteoid surface was increased possibly indicating a change in mineral metabolism. Micro-CT revealed lower cortical thickness and increased structure model index, i.e. the amount of plates and rods composing the bone trabeculas. The structural changes were paralleled by loss of function, where the null mice showed lower femoral neck failure load and tibial strength during mechanical testing at 4 months of age. The skeletal phenotype points at a role for chondroadherin in both bone and cartilage homeostasis, however, without leading to altered longitudinal growth.

## Introduction

Bone and cartilage are both made up of relatively few cells embedded in an abundant extracellular matrix (ECM). In cartilage, collagen fibrils and the negatively charged proteoglycan aggrecan, forming large aggregates with hyaluronic acid, constitute the major structural assemblies of the matrix. These two components provide tissue with tensile strength and resistance against compressive forces, respectively. The members of the small leucine rich repeat proteins (SLRPs) regulate assembly and function of the ECM, particularly the collagen networks, and include decorin, biglycan, asporin, fibromodulin, lumican, keratocan, PRELP (proline arginine-rich end leucine-rich repeat protein), osteoadherin (OSAD) and chondroadherin (CHAD) [1]. Several SLRPs have roles in bridging between cells and matrix by providing for interactions with cell surface receptors such as syndecans (CHAD and PRELP) and integrins (CHAD and OSAD) at the same time as binding to structural matrix proteins, particularly fibril forming collagens exemplified in Camper et al., 1997, Haglund et al., 2011, and Haglund et al., 2013. The important roles of the SLRP molecules in matrix organization are illustrated by the abnormalities in mice with inactivated SLRP genes showing signs of dysregulation of collagen fibril formation [2]–[5]. CHAD is a 38 kD protein, first isolated from bovine cartilage [6]. It contains 11 leucine rich repeats (LRRs) and is classified as a SLRP based on its primary structure [1]. CHAD is highly expressed in cartilaginous tissues and is primarily located close to the cells. Lower levels of expression are found in bone, tendon [6]–[8] and eye [9]. In bovine bone, CHAD is implicated in direct interaction with calcium phosphate mineral [10]. CHAD mediates adhesion of isolated chondrocytes via two mechanisms: one is binding via the α2β1 integrin [11] an interaction that can mediate signalling between chondrocytes and their extracellular matrix [12]; the other interaction is between the C-terminal chondroadherin sequence and cell surface proteoglycans such as syndecans that can act as receptors (Haglund et al., 2013). Bone CHAD promotes attachment of osteoblastic cells (Mizuno et al., 1996) and binds with high affinity to collagen types I and II [13]. Also, CHAD interacts tightly with both the N- and C-terminal globular domains of collagen type VI [14]. As CHAD can interact with structural extracellular matrix (ECM) molecules as well as with cells in the tissue, the protein may provide a mechanism for regulating cell activities in relation to ECM structure, and thus, play a role in both cartilage and bone homeostasis. CHAD has an unusually restricted tissue distribution: In rat femoral heads, CHAD is localized mainly in the territorial matrix at different stages of articular cartilage development, and CHAD mRNA is particularly prominent in the late proliferative cells in the epiphyseal growth plate at young age [15]. We now report the generation of a mouse with the CHAD gene inactivated (CHAD −/−) and have performed detailed studies of its phenotype with an emphasis on bone and cartilage homeostasis to reveal functions of CHAD *in vivo*. We found that CHAD plays roles in the cartilage development and maturation of the growth plate at young age and in the molecular composition of articular cartilage in adults as well as in bone homeostasis and function.

## Results

### 2.1. Characterization of CHAD−/− mice

CHAD null mice showed normal embryological development and appeared healthy after birth. Macroscopically no phenotypic abnormalities were visible and the mice appeared healthy up to more than one year of age. Both female and male mice were fertile and the CHAD−/− breeding pairs did not differ in litter sizes compared to WT.

### 2.2. Demonstration of gene inactivation and loss of CHAD

**Figure 1:**
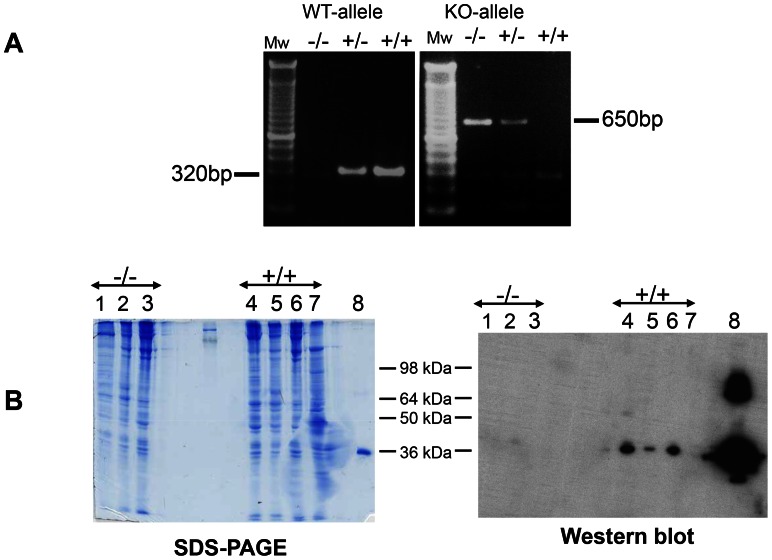
Analyses of message and proteins in CHAD−/− and WT mice. A: PCR and agarose gel electrophoresis of mouse tail samples. There is a faint, barely visible, reactivity at the position of the wild type allele (320 bp), but this is not observed in the CHAD−/− mice. It probably represents some weak reactivity of the wild type allele by the primers for the deleted allele B: Protein stained gel and Western blot of cartilages and liver as a control for non-specific reactions. Different cartilages were extracted with 4 M guanidine hydrochloride, proteins precipitated with ethanol and electrophoresed on 4–16% SDS-PAGE. Left picture represents a Coomassie stained gel and the right picture represents Western blots with the anti-CHAD antibody. The lanes represent extracts of 1. Trachea (−/−); 2. Nasal cartilage (−/−); 3. Knee cartilage (−/−); 4. Trachea (+/+); 5. Nasal cartilage (+/+); 6. Knee cartilage (+/+); 7. Liver (+/+); 8. Recombinant CHAD; −/− represents CHAD−/− and +/+ wild type mice.

In initial experiments a procedure for identification of CHAD−/− and WT mice was established by the use of PCR of tail samples prepared by routine procedures (Svensson et al., 1999) with primers selected to give different products when CHAD was present or not. These products were distinguished by Agarose gel electrophoresis (fig. 1a). WT mice demonstrated one band of 650 bp while the null mice showed an expected band of 320 bp and the heterozygote showing both bands. The data clearly demonstrate disruption of the CHAD gene in the null animals. This was further substantiated by Western blotting confirming the absence of CHAD in extracts from the null mice as compared to wild-type cartilage, which showed robust expression of CHAD. Liver tissue, which does not normally express CHAD, was used as an additional negative control to confirm that the antibody was not recognizing non-specific bands (fig. 1b).

### 2.3. General morphology

Heart, lung including bronchial cartilage, kidney, liver and spleen showed no histopathological changes by systematic investigation at the light microscopic level of paraffin sections. Since chondroadherin is expressed in the eye, a more detailed study by semi thin epon sections was undertaken but showed no differences (data not shown).

### 2.4. Tissue screening by DXA scanning

BMD, lean and fat content were measured in mice 6 weeks, and 3, 5 and 8 months of age. Results showed only very small differences in 6 week-old males lacking CHAD compared to controls. In this group the BMD/mg body weight was slightly lower (CHAD−/−  = 1.79±0.1 mm−2, WT  = 1.88±0.4 mm−2, p = 0.03), so was the fat content measured in the whole mouse (CHAD−/−  = 11.04%±0.26, WT  = 12.23%±0.40, p = 0.03). Apart from these differences, the DXA data did not reveal any abnormalities in CHAD-null mice (data not shown).

### 2.5. Cartilage

#### 2.5.1. The epiphyseal growth plate

**Figure 2:**
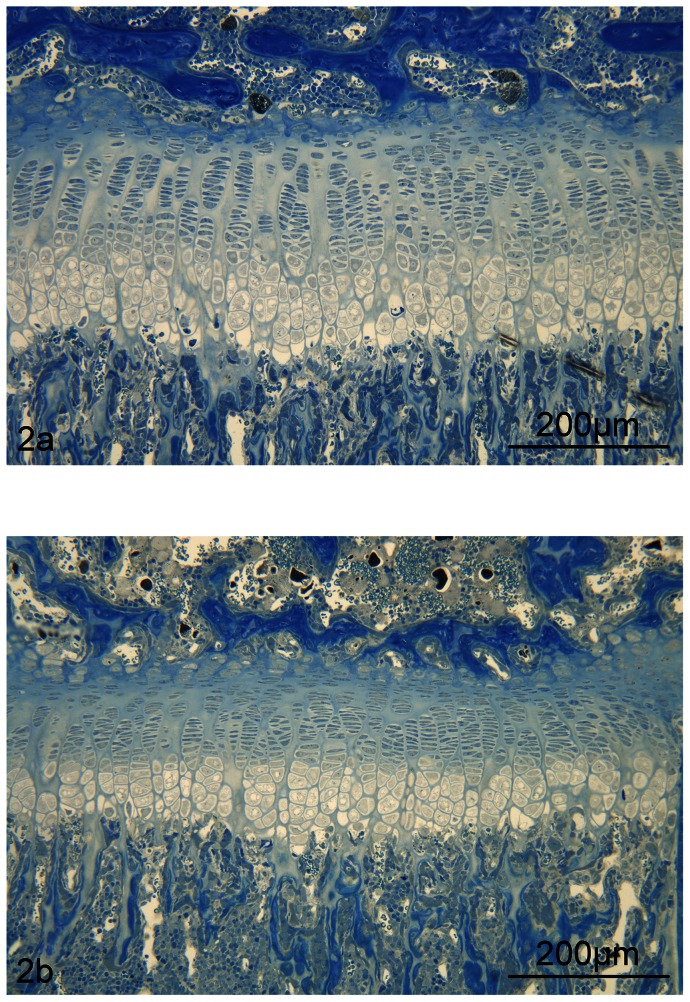
Micrographs of the epiphyseal plate in CHAD −/− and WT mice. Light micrographs of CHAD−/− (2a) and WT (2b) mice at 3 weeks of age. A small but significant increase in the height of the growth plate mostly confined to the proliferative zone was confirmed by histomorphometry (table 3). Epon-embedded tissue, toluidine blue staining (x 20).

Overall the CHAD−/− mice presented a 35% increase in mean height of the femoral epiphyseal growth plate at 3 weeks of age (p = 0.02, table 1), despite normal length of the femur. When the relative height of each zone was calculated, the resting zone was increased by 30% and the proliferating zone by 45% in CHAD−/− mice aged 3 weeks (p = 0.04 and p = 0.007, respectively) (fig. 2). At 6 weeks of age the proliferating zone was increased by 20% compared to WT mice (p = 0.04).

**Table 1:** Histomorphometric analyses of the epiphyseal growth plate in 3 and 6 weeks old CHAD−/− mice and age-matched WT mice.

		3 weeks old		6 weeks old	
	Zone	CHAD−/−	WT	CHAD−/−	WT
Epiphyseal height	–	288±54 *	214±14	269±31	238±44
Height of zone	Resting	39±8 *	30±4	32±3	37±9
	Proliferating	155±27 **	107±6	151±16 *	126±23
	Hypertrophic	94±23	77±8	85±15	74±14
Osteoid surface	Metaphysis	–	–	21±6	17±5

Values are mean ± SD. For epiphyseal values (µm), n = 6/4 and n = 6/8 for CHAD−/− and WT mice after 3 and 6 weeks, respectively. For osteoid values (% of trabecular surface), n = 7/6. *p<0.05, ** p<0.01.

#### 2.5.2. Expression and localization of proteins in growth plate cartilage

We analysed the expression and organization of a number of proteins in the growth plate to discern differences in the tissue organization upon CHAD inactivation. In both null and wild type animals the distribution of mRNA showed the expected pattern of expression: Cartilage oligomeric protein (COMP) mRNA was primarily detected in the proliferative chondrocytes of the epiphyseal growth plate, with no significant difference between the groups. Osteopontin (OPN) and BSP were both detected in hypertrophic chondrocytes, while there was no detectable difference. Immunostaining for COMP was almost exclusively localized to the epiphyseal growth plate, in addition to some cartilaginous remnants in trabecular areas. The staining was most intense in the territorial matrix along the columns of proliferative chondrocytes, although pericellular staining was observed to various degrees in all zones, and weaker staining was observed in the interterritorial matrix ( fig. S1). Histological scoring of COMP staining did not demonstrate any differences between CHAD−/− and WT mice (data not shown).

#### 2.5.3. Changes in the proteome of articular cartilage

Initially there were 226 proteins identified by MASCOT 2.1 from 5457 peptide matches above homology or identity threshold. After filtering the data removing obvious false positives (13 proteins) and protein hits with only one peptide hit, it was possible to measure and calculate the relative ratios of 178 proteins detected in all extracts (table S1). The proteins identified include the major components of the extracellular matrix in cartilage such as collagens, aggrecan and members of the SLRPs family. The comparisons were made without normalization although the average ratios for all proteins were 0.8–0.9 suggesting that the total protein content of the samples from the knockout animals may be slightly lower than the corresponding wild type samples. Overall most of the proteins were present at the same level in the null compared with the WT mice. However, some differences were noted (table 2). The null CHAD mouse vs. its wild type counterpart showed increased levels of fibronectin (ratio 1.85 and 1.61 vs wild type). Both secreted parts of the gene product of CILP 1 (CILP 1–1 and 1–2) were particularly elevated in female null mice (ratio of 1.84 and 1.63, respectively). Markedly decreased levels in the null mice were noted for alpha-1-antitrypsin 1 family members (ratio around 0.3) and apolipoprotein E. Expectedly, CHAD in the null mice were at background noise levels. In support no CHAD was detected in LC-MS analyses of the individual samples of the CHAD null mouse. Corresponding western blots showed no reactivity at all verifying that the sample preparation is free from cross-contamination. Most other proteins showed similar levels in null and wild type mice.

**Table 2:** Changes in the proteome of cartilage from mice with the CHAD gene inactivated.

Acc. No.	Protein name	Fem KO vs Wt	Male KO vs Wt
P11276	Fibronectin	1,85	1,61
Q66K08	Cartilage intermediate layer protein 1	1,75	1,18
P31725	Protein S100-A9	1,02	0,21
Q9Z1F6	Chondromodulin-1	0,97	0,49
P29699	Alpha-2-HS-glycoprotein	0,82	0,46
P62259	14-3-3 protein epsilon	0,68	0,33
P63101	14-3-3 protein zeta/delta	0,66	0,32
P22599	Alpha-1-antitrypsin 1-2	0,39	0,27
Q00898	Alpha-1-antitrypsin 1-5	0,38	0,28
Q00897	Alpha-1-antitrypsin 1-4	0,38	0,27
Q00896	Alpha-1-antitrypsin 1-3	0,38	0,25
P07758	Alpha-1-antitrypsin 1-1	0,37	0,25
P08226	Apolipoprotein E	0,36	0,35

Acc. No.	Protein domains	Fem KO vs Wt	Male KO vs Wt
P28481	Collagen alpha-1(II) chain	1,23	1,40
P28481	Collagen C-propeptide	0,90	0,81
P28481	Collagen N-propeptide	1,00	1,37
Q66K08	Cartilage intermediate layer protein 1-2	1,84	1,27
Q66K08	Cartilage intermediate layer protein 1-1	1,63	1,06
D3Z7H8	Cartilage intermediate layer protein 2-2	1,46	1,08
SLRP proteins			
Q99MQ4	Asporin	*0,90*	*1,11*
P28653	Biglycan	1,16	1,11
P28654	Decorin	1,00	1,05
P70186	Epiphycan	0,84	0,90
P50608	Fibromodulin	1,01	1,05
P51885	Lumican	0,98	0,92
Q62000	Mimecan	0,80	0,89
O35103	Osteomodulin	0,77	1,02
Q9JK53	Prolargin	1,10	1,17

### 2.6. Bone

#### 2.6.1. Micro-CT

Screening of mice aged 5 days, 3 week and 4 months showed that the length of the femora (i.e. the distance between the distal growth plate and the gluteal tuberosity) increased with time but was not significantly different between CHAD−/− and WT mice (table S2).

The 4 months old mice were analysed further. A number of parameters expectedly showed significantly higher values for the male wild type mice (table 3). Particularly noticeable differences were the higher trabecular thickness and structure model index for the null animals (p<0.05). There were also some noticeable differences in the form of lower polar moment of inertia and cortical diameter at midshaft only apparent for the male null mouse (p<0.05), where values were more similar to those of the females. The value for trabecular spacing in the female null mice was lower than the wild type and more similar to those of the male, although values did not reach significance.

**Table 3:** Micro-CT cortical/trabecular bone parameters in 4 month old mice.

Parameter	Male KO	Male WT	Female KO	Female WT	Average KO	Average WT
Bone length (mm)	7.04±0.36	6.82±0.52	6.75±0.28	7.27±0.35	6.89 ±0.34	7.10±0.45
Ct.Th (mm)	0.18±0.01	0.17±0.02^c^	0.18±0.01^b^	0.19±0.01	0.18±0.01	0.18±0.02
vBMD (g/cm^−3^)	1.57±0.08	1.51±0.04^c^	1.55±0.08	1.59±0.06	1.56±0.08	1.56±0.07
Ct.BV (mm^3^)	99.61±0.58	99.66±0.17^ c^	99.62±0.44	99.82±0.07	99.61±0.50	99.76±0.14
Ct.P (%)	0.39±0.58	0.34±0.17^ c^	0.38±0.44	0.18±0.07	0.39±0.50	0.24±0.14
Tb.BV (%)	7.43±1.37	7.43±1.44^ d^	5.13±2.27	4.35±0.69	6.28±2.13	5.51±1.85
Tb.P (%)	92.57±1.37	92.57±1.44^ d^	94.87±2.28	95.65±0.69	93.72±2.13	94.49±1.85
Tb.Th (mm)	0.046±0.003	0.042±0.005	0.043±0.004	0.041±0.002	0.045±0.003^a^	0.041±0.003
Tb.Sp (mm)	0.27±0.01	0.27±0.01^c^	0.29±0.02	0.33±0.03	0.28±0.02	0.31±0.04
DA	2.14±0.20	2.16±0.13	2.24±0.35	2.56±0.54	2.19±0.27	2.41±0.46
SMI	2.43±0.11b	2.23±0.01^c^	2.53±0.36	2.42±0.07	2.48±0.25^a^	2.34±0.11
MMI (mm^4^)	0.33±0.02^a^	0.72±0.15^d^	0.34±0.06	0.35±0.04	0.33±0.04	0.49±0.21
D (mm)	0.97±0.01^a^	1.15±0.06^d^	0.99±0.04	0.99±0.02	0.98±0.04	1.05±0.09

Bone length, cortical/trabecular thickness (Ct.Th/Tb.Th), volumetric BMD (vBMD), cortical/trabecular porosity (Ct.P/Tb.P), trabecular separation (Tb.Sp), degree of anisotropy (DA), structure model index (SMI), polar moment of inertia (MMI) at midshaft, and equivalent circle diameter (D) at midshaft of femur in CHAD−/− (KO) and WT mice. ^a^: p≤0.05 KO vs. WT, ^b^: p≤0.05 KO male/female vs. WT male/female, ^c^: p≤0.05/^d^: p≤0.01 males vs. females in same group. Taken together the data clearly demonstrate an altered bone homeostasis in the mice with the chondroadherin gene inactivated.

#### 2.6.2. Collagen fibres in bone

Qualitative electron microscopic analysis of 6 weeks old CHAD−/− mice showed no abnormalities in the structure, tissue organization and thickness of collagen fibrils in calvarial bone compared to WT mice (data not shown).

#### 2.6.3. Protein expression in bone

Tartrate resistant acid phosphatase (TRAP) mRNA was primarily detected in metaphyseal osteoclasts; in addition, some resting and hypertrophic chondrocytes in the growth plate also expressed TRAP. Cathepsin K (CTK) mRNA was solely present in multinucleated cells in the metaphyseal region. OPN mRNA was detected primarily in osteoblasts lining the metaphyseal trabecular surfaces. As for OPN, BSP mRNA was highly expressed in osteoblasts lining trabeculae.

**Figure 3:**
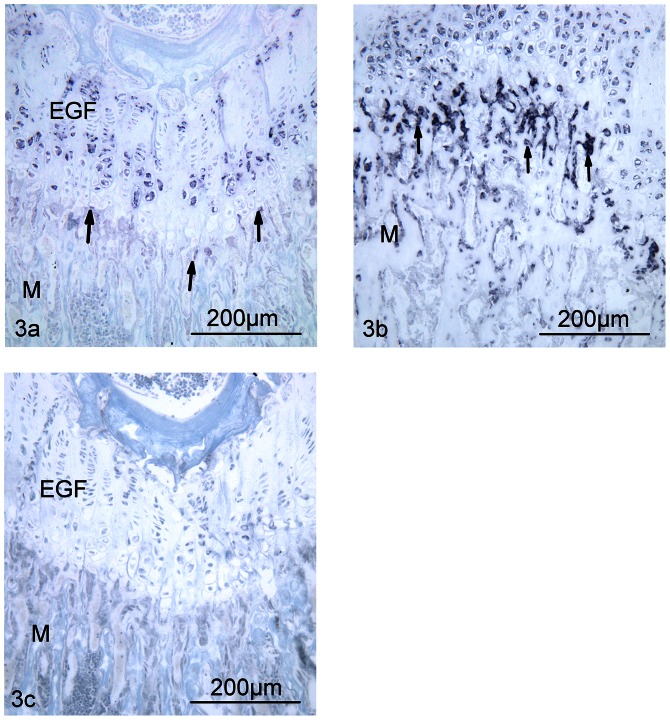
BSP mRNA expressing cells in the distal femur of CHAD−/− and WT mice at 6 weeks of age. *In situ* hybridization showed intense signal in multiple osteoblastic cells (arrows) in the metaphysis (M) of WT mice (3b), while CHAD−/− mice (3a) showed very sparse signal in cells in the corresponding area (p = 0.01). Also chondrocytes in the epiphyseal growth plate (EGF) showed signal, although no quantitative difference in number of cells were detected between the groups at this site (x 20). Negative control with sense probe was without signal (3c).

The relative number of BSP mRNA expressing cells was significantly lower in the metaphysis of CHAD−/− mice compared with WT (p = 0.01) (fig. 3). Thus, the mean score in the metaphysis for null mice was 0.4 (SD 0.9) as compared to WT 1.8 (0.4); n = 5 in both groups. No differences were found for relative number of COMP, TRAP, CTK or OPN mRNA expressing cells (data not shown).

#### 2.6.4. Protein localization in bone

The most intense accumulation of gold particles for both BSP (fig. S2) and OPN was observed at electron dense extracellular areas representing osteoid-bone interface/mineralization fronts, and to a lesser extent, diffusely spread in mineralized bone. BSP exhibited a characteristic pattern with labelling confined to discrete sites in bone matrix corresponding to areas of early mineral deposition. Semi-quantitative analysis revealed a trend towards increased signal intensity in most compartments for both proteins in CHAD−/− mice (table 4). However, when each compartment was compared between CHAD−/− and WT mice, only BSP labeling in osteoid was found to be significantly increased (table 4).

**Table 4:** Ultrastructural distribution of OPN and BSP in bone of CHAD−/− and WT mice.

	OPN		BSP	
Compartment	CHAD−/− (n = 6)	WT (n = 6)	CHAD−/− (n = 6)	WT (n = 6)
OB nucleus	1.53±0.46	1.32±0.41	0.94±0.19	1.08±0.19
OB cytoplasm	1.69±0.38	1.70±0.36	0.82±0.15	0.72±0.18
Osteoid	4.54±2.50	3.70±2.06	1.20±0.21*	0.93±0.14
Mineralization front	150.18±51.42	124.21±56.63	16.51±5.60	15.35±6.45
Mineralized bone	15.96±12.40	9.30±6.77	3.83±0.88	2.98±0.68
Capillary lumen	0.80±0.22	0.57±0.20	0.46±0.07	0.50±0.15

Semi quantitative analysis of the ultrastructural protein distribution in CHAD−/− and WT mice. Values are mean ± SD gold particles/um^2^ per animal. OB  =  osteoblast. * p<0.05 when compartments are directly compared.

#### 2.6.5. Mechanical properties of bone

**Figure 4:**
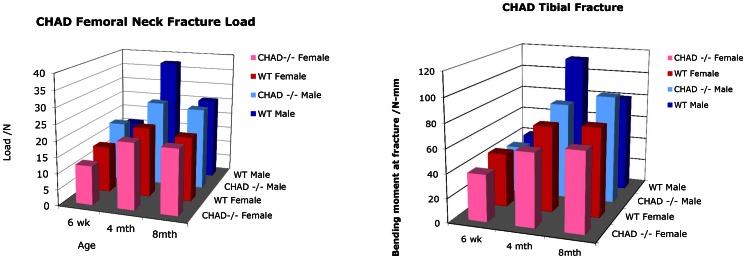
Mechanical properties of bone. 4a: Femoral neck failure load (Newtons) for 6 weeks, 4 months and 8 months old wild type (wt) and CHAD−/− male and female mice. The difference between CHAD−/− and wt in 4 months old male mice is significant (p<0.01). 4b: Tibial shaft failure load (Newtons) for 6 weeks, 4 months and 8 months old wild type (wt) and CHAD−/− male and female mice. The difference between CHAD−/− and wt in 4 months old mice is significant for both genders (p<0.001).

All mechanical properties increase significantly from 6 weeks to 4 months of age (fig. 4). Femoral neck failure load (fig. 4a) was significantly lower in the 4 month old CHAD−/− mice compared to the same age and gender wild type (p<0.01). The difference between CHAD−/− and wild type female at 4 months was small and not significant although again the wild type may show somewhat higher bone strength. Males showed higher strength than females. In contrast at 8 month there were no observable differences between the mice whether wild type-mutant or female-male were compared. Interestingly the strength of the 4 months wild type male mice appeared higher than that of 8 months animals. The tibial strength (fig. 4b) showed similar trends to the femoral neck strength. The null mice showed significantly lower strength than the wild type at 4 months both for males and females (p<0.001). At 8 months differences could not be discerned.

## Discussion

CHAD deficient mice did not show gross anatomical defects, grew to normal size, were fertile, and had a normal life span up to 2 years of age, which is in line with other studies with SLRPs-null mice [2]–[5]. However, the CHAD deficient mice presented a distinct skeletal phenotype, demonstrating a role for CHAD in cartilage and bone turnover. With the exception of increased levels of CILP-1 and fibronectin in the female CHAD deficient mouse, alterations in the identifiable extracellular matrix proteins proper in articular cartilage were small. The altered levels of both CILP proteins in particularly the female mice, albeit still only by some 50%, are interesting but at this time the functional implications are not known. It can be noted that CILP is a protein up regulated in osteoarthritis (Bernardo et al., 2011), and there is a polymorphism that correlates to a higher incidence of lumbar disc disease (Seko et al., 2005). The mice at the ages studied showed no signs of either joint or spine disease. An unexpected finding was the consistently low levels of several variants of alpha-1-antitrypsin. This might affect the susceptibility against proteolytic activity and thereby also overall tissue stability. Interestingly, it is rather obvious that none of the other SLRP proteins are differently expressed following the removal of CHAD.

### 3.1. Disturbances in the epiphyseal growth plate but normal collagen organization

The CHAD −/− mice presented a widened epiphyseal growth plate. This was most pronounced in the proliferative zone at 3 and 6 weeks of age which fits well with data showing that CHAD is synthesized mainly by late proliferative chondrocytes [7]. The balance between proliferation and differentiation of chondrocytes is an important regulatory step controlled by multiple signalling molecules, including the Indian hedgehog (Ihh)/parathyroid hormone related peptide (PTHrP) feedback loop [16]. Interestingly, Ihh, which is upstream in the signalling pathway of PTHrP, shows a similar distribution of expression to that of CHAD [17]. Ihh controls the transition from proliferating to hypertrophic chondrocytes [16], thereby regulating the height of the proliferative zone. Thus, based on the very distinct localization around a portion of the proliferative chondrocytes, the apparent absence of cell spreading and growth of chondrocytes on CHAD coated surfaces [18], as well as the observed widening of the proliferative zone in null mice, it could be speculated that CHAD may influence the Ihh/PTHrP feedback loop and/or participate in the control of chondrocyte development by promoting their differentiation into the hypertrophic stage. However, the lack of detectable differences in bone length between null and WT mice suggest that CHAD influences chondrocyte maturation only to a modest degree or that other processes compensate by modulating subsequent events. It has been documented that CHAD interacts with collagen and the protein is abundant in the territorial matrix, suggesting a role in early assembly and function of fibrillar collagen [13]. However, CHAD-null mice showed normal collagen organization and fibril diameter in the bone, indicating either that other molecules than CHAD play more prominent roles in the process or that CHAD differs from other SLRPs not only with respect to localization, but also regarding its function *vis-à-vis* collagen [4].

### 3.2. Altered mechanical properties, cortical/trabecular bone parameters and loss of sex-specific differences

Significant differences in trabecular/cortical parameters were apparent at the age of 4 months, where the null mice presented significantly higher BMD, lower cortical thickness, increased trabecular thickness, and increased structure model index (SMI) by micro-CT. SMI is a measure of the ratio of “plate-like” to “rod-like” trabecula within a trabecular bone specimen and higher density cancellous bone generally shows more “plate-like” trabecula. Interestingly, osteoporotic trabecular bone transits from plate-like to rod-like, increasing the SMI [19]. Thus, increased SMI of the trabecular bone in the null mice is consistent with impaired mechanical properties. In line with the micro-CT data indicating disturbed formation and/or remodelling of bone, the mechanical testing showed reduced mechanical strength of both femoral neck cancellous bone and tibial cortical bone at 4 months. This was most pronounced for the male mice, possibly reflecting different rates of bone turnover between male and female. The older 8 months mice showed no such discernable difference, perhaps indicating a lower bone metabolism at this age. Noteworthy, this group of mice was based on the C57BL/6 strain which has been shown to have larger cortical cross-section areas but to be less responsive to increased mechanical loading than other used strains, e.g. the C3H/He and DBA/2 (Robling et al., 2002). Our data show that CHAD influences both cortical and trabecular bone formation and/or remodelling. The male null mice showed an appearance of the studied variables more similar to the parameters observed for the wild type female mice. Our data suggest that CHAD is important in the sex-specific development of the skeleton. Such loss of sex-specific differences has previously also been reported in OPN deficient mice [20].

### 3.3. Decreased number of cells expressing BSP mRNA

Non collagenous proteins of the SIBLING (small integrin-binding ligand, N-linked glycoprotein) family (Fisher et al., 2001), which includes OPN and BSP, are believed to play key biological roles in the development, turnover and mineralization of bone (reviewed in [21] and [22]). Both BSP and OPN are secreted by osteoblasts and have been shown to modulate osteoblast differentiation and mineralization *in vitro.* BSP for the most part promotes the process [23]–[25]. Interestingly, a considerable decrease in the number of BSP mRNA expressing cells was noted in the CHAD null mice. This decrease together with the slightly increased osteoid surface observed in the femoral metaphysis of these mice may imply impaired mineralization. On the background of altered cortical/trabecular parameters and decreased number of BSP mRNA positive cells in the distal femur metaphysis of CHAD-null mice, we extended the study of BSP and OPN and investigated their protein distribution in bone at the ultrastructural level. Despite a tendency towards increased signal intensity in osteoid, over osteoid-bone interfaces/mineralization fronts as well as in mineralized bone for both proteins in CHAD-null mice, there was no overall significant difference in the protein distribution pattern.

However, this observation is not necessarily contrary to the *in situ* hybridization data, as protein distribution in the tissue depends not only on synthesis but also on secretion and degradation in the ECM. Thus, although there are fewer cells expressing BSP mRNA in CHAD-null mice, the protein synthesis of those expressing the gene appears normal, and the number of cells expressing OPN is normal. Thus, taken together, the CHAD null mouse appears to have an altered and lower bone turnover.

## Conclusions

The present study has provided the first evidence that the absence of CHAD leads to a distinct skeletal phenotype characterized by widening of the epiphyseal growth plate with possible impaired of hypertrophic differentiation of chondrocytes, reduced number of BSP expressing cells, disturbed molecular composition of articular cartilage and structural and functional alterations in trabecular and cortical bone tissue with alterations in bone turnover.

## Materials and Methods

This study was carried out in strict accordance with the institutional guidelines for animal research at Lund University, Sweden. The protocols were approved the Committee on the Ethics of Animals at Lund University, Sweden (Permit Numbers: M31-09 and M177-11).

### 5.1. Generation of CHAD−/− mice

A mouse genomic cosmid library was screened using a 887-bp CHAD rat cDNA fragment as described [26]. A 31 kbp genomic DNA fragment was isolated and partly sequenced. Out of this a 3000 bp fragment including the ATG of the CHAD gene was inserted to the pWH9 vector (kindly provided by Dr. R. Fässler) that carries a phosphoglycerate kinase-neomycin resistance cassette (pGKNeo). The 3000 bp fragment was inserted 5′ of the neocassette. In the 3′end of the cassette a 7000 bp CHAD fragment was inserted.

#### 5.1.1. Generation of recombinant ES cell lines and chimeric mice

Approximately 20×106 semi-confluent R1 embryonic stem (ES) cells (kindly provided by professor Reinhard Fässler) (Nagy et al., 1993) were electroporated with 80 µg linearized targeting vector. The ES cells were cultured on feeder cells in DMEM supplemented with fetal calf serum and leukemia inhibitor factor (for reference see[27]). After 24 hours of culture, selection for positive clones was initiated by the addition of 500 µg/ml G418. Positive clones were picked, expanded and DNA was purified and subsequently analyzed by Southern Blot analysis for confirmation of the correct targeting events. Targeted ES-cells were injected into mouse blastocysts according to standard procedures. Chimeric males were mated with C57BL/6 females and males with germ line transmission were further bred with 129/sv females to establish a strain of CHAD-null mice. Before analyses, the mice were backcrossed for 10 generations into the C57BL/6 background.

#### 5.1.2. Genotyping of CHAD−/− mice

Genomic DNA from tail tendon was purified, digested with EcoR1 and separated on an agarose gel using standard procedures. A 1000 bp XbaI-HindIII fragment was used as a probe in the hybridization. This probe detects a 16 kbp fragment in the wild type (WT) mouse and a 13 kbp fragment in the targeted mouse.

PCR was used to detect homologous recombination of the mouse CHAD gene. A 5′primer 5′CAG TCT GGT CTT TCT TGC CA was used together with a 3′primer 5′ATG TCG TTG TGG GAC AGG TA. This detects a 320 bp fragment in the WT mouse. An additional primer corresponding to the sequence 5′CGC CTT CTT GAC GAG TTC TT in the neo-cassette was used to detect a fragment of 650 bp corresponding to homologous recombination in the knock-out.

### 5.2. Skeletal X-ray analysis

Bone mineral density (BMD), fat and lean content were examined with dual-energy X-ray absorption (DXA) using the Lunar PIXImus Densitometer (GE Medical Systems). Measurements were performed on null and WT mice at the age of 6 weeks, and 3, 5, and 8 months of age (males and females separately). At each time-point at least 6 CHAD−/− and 6 WT animals were measured. The measurements were performed on anaesthetized living animals.

### 5.3. Micro computed tomography

Micro computed tomography (micro-CT) was performed as two experiments: first, femora of mice sacrificed at 5 days, 3 weeks and 4 months of age, respectively, were included as a general screening. In the second experiment, in depth analyses were performed in both sexes of 4 months old mice (4 male and 4 female CHAD−/−, and 5 male and 3 female WT mice). All specimens were scanned by the use of high-resolution micro-CT (SkyScan 1172; SkyScan, Kontich, Belgium). Dissected whole femora were affixed to the scanning stage and projection images were obtained at a resolution of 8.03 µm and reconstructed by use of manufacturer-provided software (NRecon, SkyScan). After calibration of the standard unit of X-ray CT density (Hounsfield unit, HU), the conversion from HU to volumetric bone mineral density (vBMD) was done. Reconstructed images were analyzed by use of manufacturer-supplied software. Three sections as shown in fig. S3 consisting of 63 slices or 0.5006 mm (5 days old mice) or 126 slices or 1.012 mm (3 weeks and 4 months old mice) were analyzed per bone for the following parameters: cortical thickness, cortical porosity, cortical bone volume, trabecular thickness, trabecular separation, trabecular bone volume, trabecular porosity, as well as degree of anisotropy (DA) (from mean intercept length analysis as an index of degree of preferred orientation of the structure [28]) and structure model index (SMI) (the amount of plates and rod composing the structure [19]). A threshold of 45, 66, and 86 to 255 was applied for 5 days, 3 weeks and 4 months old mice, respectively (fig. S4). Cortical vBMD was obtained after applying a threshold of 1–255 to sections of cortical bone.

### 5.4. Macroscopic and light microscopic analyses

At sacrifice the mice were subjected to macroscopic work up aiming at detection of malformation. Moreover, samples from heart muscle, kidney, spleen, liver and lung were fixed in formalin, paraffin-embedded, sectioned and stained with haematoxylin & eosin (H&E) according to a routine protocol. Intact eyes were fixed in 2% glutar aldehyde and embedded in an epoxy resin (Epon 812, Agar Scientific ltd., Stansted, Essex, UK) and equatorial semi thin sections were stained with toluidine blue according to a routine protocol. Three to five coded sections per organ and animal were subjected to conventional light microscopy by an experienced surgical pathologist and evaluated for structural tissue changes. Six animals (CHAD−/− and WT) were investigated at each age, i.e. 3 and 6 weeks as well as 3 and 8 months.

#### 5.4.1. Bone histomorphometry

Femora from 3 and 6 weeks old animals were fixed in glutar aldehyde, decalcified in 7% EDTA for 15 days and embedded in an epoxy resin as above. Semi thin longitudinal sections of distal femoral metaphyses were cut and stained with toluidine blue, and histomorphometric analysis was performed on digital images (resolution 2576×1932 pixels) using image analysis software (AnalySIS pro, Digital Soft Imaging System, Münster, Germany). Mean height of the epiphyseal growth plate was calculated for each section using the mean of 10 randomly placed lines for measurement. The relative zonal distribution of the resting, proliferative and hypertrophic zone was estimated by point counting.

Femora for measurement of osteoid were fixed in 4% buffered formalin, embedded in a methyl methacrylate resin (K-plast, DiaTec Systems, Germany) without prior decalcification, sectioned and stained with Masson-Goldner's trichrome. Relative osteoid surface (% of trabecular surface) was estimated by point counting. For each animal, a minimum of 3 non-overlapping visual fields of vision were analyzed.

#### 5.4.2. *In situ* hybridization

Five to six 6 weeks old animals from each group were subjected to *in situ* hybridization with riboprobes for OPN, CTK, BSP, TRAP and COMP. Gene sequences for TRAP, CTK, COMP, OPN and BSP were amplified by conventional PCR using cDNA from mouse osteoblasts (a generous gift from dr. Rune Jemtland, Oslo University Hospital, Norway) or IMAGE clones using the oligonucleotide primers listed in table S3. All sequences were subsequently cloned with a Dual Promoter TA Cloning Kit (Invitrogen) and sequenced. Digoxigenin (DIG)- conjugated complementary RNA cRNA) probes were synthesized with a DIG-labelling kit (Roche Diagnostics AS, Oslo, Norway) using T7 or Sp6 RNA polymerase to yield probes in the sense or antisense orientation. Hybridization of longitudinal sections of formalin-fixed femora embedded in paraffin was performed by modification of a previously described protocol [29]. Briefly, dewaxed and proteinase K-digested sections of paraffin-embedded samples were post-fixed in paraform aldehyde. Following prehybridization in formamide/2× SSC, the sections were hybridized with 5 ng probe in 50% formamide/2× SSC/7.5% dextran sulphate. High stringency washing was performed, and unbound probe was removed by RNase-treatment. Hybridized probe was detected using an alkaline phosphatase (AP)-conjugated sheep anti-DIG antibody followed by the AP-substrate nitrobluetetrazolium chloride (NTB)/5-bromo-4- chloro-3-indolyl-phosphate (BCIP) (Roche Diagnostics GmbH, Mannheim, Germany). Coded sections of the epiphyseal growth plate and the metaphysis of the distal femur were micrographed and analyzed focusing on the resting zone, the proliferative zone, the hypertrophic zone, and the metaphysis. The following scoring system was used to semi-quantify mRNA positive cells; 0 =  no positive cells, 1 =  low concentration of positive cells, 2 =  high concentration positive cells.

#### 5.4.3. Immunohistochemistry

Ten 6 week old animals from each group were included in the analysis. Immunohistochemistry of COMP was performed using the peroxide technique with diaminobenzidine (DAB) as the chromogen according to a routine protocol (Hect et al., 2004). Longitudinal sections of formalin-fixed femoral bone sections embedded in paraffin were used. Following permeabilisation by digestion with chondroitinase ABC (Seikagaku Corporation, Tokyo, Japan) in tris/acetate buffer, the sections were incubated with rabbit polyclonal antiserum raised to rat COMP [30]. Bound antibodies were visualized using the Dako EnVision+ System (EnVision+ System, HRP K4010, DAKO, USA). The sections were counterstained with haematoxylin and subsequently with a mixture of eosin and phloxine B. COMP-staining was confined to the articular cartilage and the epiphyseal growth plate, and the latter was subjected to semi – quantitative analysis. Thus, the growth plate was divided into the following zones; I (resting and proliferative zones) and II (hypertrophic zone). In zone I, scores (0 =  no staining, 1 =  weak staining and 2 =  intense staining) for territorial matrix and interterritorial matrix were analyzed, while in zone II, pericellular, interterritorial and intracellular staining were graded.

### 5.5. Transmission electron microscopy

Tibias from 6 weeks old mice were immediately dissected free and fixed by immersion in a solution of 2% paraform aldehyde and 0.5% glutar aldehyde (GA). Subsequently, the tissue was embedded at low temperature in a freeze substitution device according to our established protocol [31].

#### 5.5.1. Qualitative ultrastructural collagen analysis

Coded ultrathin sections from GA-fixed, epon-embedded samples of calvarial bone of 6 weeks old CHAD −/− and WT mice were subjected to electron microscopy of collagen fibrils. The fibrils were evaluated semi-quantitatively for thickness and spatial orientation. Sections from two blocks of each of 6 animals (3 CHAD−/− and 3 WT) were investigated and categorized as normal or pathological.

#### 5.5.2. Immunogold labelling and semi-quantitative analysis

Immunogold labelling with antibodies against BSP and OPN was performed as previously described [32]. Micrographs were obtained by systematic random sampling of cells/surrounding matrix and analyzed using the semiautomatic interactive image analyzer software AnalySIS® pro (Soft Imaging System, Münster, Germany). In consensus with previous reports of the ultrastructural distribution of BSP [33], [34] and OPN [31], [35], regions of interests were confined to 1) osteoblast nucleus, 2) osteoblast cytoplasm, 3) osteoid, 4) osteoid-bone interface/mineralization fronts and 5) mineralized bone. Six animals from each group were included in the analysis, and 2 tissue blocks were sampled per animal. The results for OPN and BSP are based on the analysis of 60 osteoblasts and their surrounding microenvironment in each group.

### 5.6. Protein contents of femoral head cartilage by proteomics

#### 5.6.1. Dissection, pulverization and protein extraction of cartilage

Samples were obtained from G3 mice 4 months old. Full thickness femoral head cartilage was dissected from 8 female CHAD −/−, 7 female wild type, 7 male CHAD −/−, and 7 male wild type. The tissue from each group was pooled separately and homogeneous powder was made in liquid nitrogen. The samples were extracted with guanidine hydrochloride with added proteinase inhibitors (Larsson et al., 1991) and extracts were collected after centrifugation (IEC Micromax) at 13200 rpm for 30 min.

#### 5.6.2. Preparation of proteins in extracts for quantitative proteomics and analyses

Procedures for quantitative proteomics were the same as those described for the analyses of a set of human cartilage tissues. Essentially, proteins in extracts were reduced and alkylated, followed by trypsin digestion (Onnerfjord et al., 2012). An isobaric 4-plex ITRAQ^TM^ was then used to enable simultaneous analysis of a mix of all the four samples. Trypsin digests of the four pools of cartilage were separately labelled using standard protocols according to the manufacturer. The trypsin digested labelled extracts were combined and chromatographed on a SCX cation exchange column. The 29 fractions collected were separately applied to and analyzed using a reversed phase C18 nano-LC column online with a QTOF mass spectrometer as described (Onnerfjord et al., 2012).

#### 5.6.3. Database searching

The mass spectrometric raw data was processed using Protein Lynx 2.1 with internal calibration. The processed files were searched with taxonomy mus musculus using MASCOT 2.1. The ratios of individual peptides between female CHAD −/− vs. wild type, male CHAD −/− vs. wild type, female CHAD−/− vs. male CHAD −/−, and female wild type vs. male wild type mice were calculated by MASCOT.

#### 5.6.4. Data analysis

iTRAQ quantification parameters: significant threshold p<0.05; weighted average ratios; minimum number of peptides of 2, minimum precursor charges of 2; at least homology of 0.05. The searched list was manually inspected for errors and a limited number of obviously incorrectly identified proteins were removed e.g. non-collagenous proteins identified with hydroxylation on proline residues.

### 5.7. Analysis of tissue protein pattern by SDS-polyacrylamide gel electrophoresis and presence of CHAD by Western blot

Cartilage from the femoral head, trachea and nose were dissected clean, cut into small pieces and extracted with guanidine-HCl (GuHCl) containing a proteinase inhibitor cocktail according to standard procedures [6]. For control of antibody specificity in the Western blot a liver sample was treated in the same way. Samples of extracts corresponding to 1 mg of wet weight tissue were precipitated with ethanol and electrophoresed on SDS-polyacrylamide 4–16% gradient gels followed by Western blotting as described [6], [36]. The antibody used to stain the blot was raised in rabbits against bovine CHAD [6].

### 5.8. Testing of mechanical properties of bone by fracturing

Mice were sacrificed at 6 weeks, 4 or 8 months of age and frozen at −20°C. At the time of testing the mice were thawed, the femora and tibiae were dissected out and kept wet being wrapped in saline soaked tissue at 4°C overnight prior to testing. Two different mechanical tests were performed aimed at measuring femoral neck for the properties of cancellous bone and tibia for cortical bone. The strength of the cancellous bone of the femoral neck was measured using a miniaturised version of the mechanical test previously developed to test total hip replacements (Thompson et al., 2004). The bone was gripped in a cylindrical holder, which was then held at 9° to the vertical, orientated so that the bone was vertical in the sagittal plane and in valgus in the frontal plane, thus similar to the position of the femur *in vivo*. Using an Instron® 8511.20 biaxial load frame with an MTS® TestStar II controller, displacement was applied to the femoral head, using a flat ended indenter, at 0.1 mm s^−1^ until fracture occurred. Care was taken to ensure that the load was applied to the top of the femoral head, such that the loading indenter was not touching the greater trochanter. Fracture lead to a drop in the applied load. After removal from the mechanical test machine the specimen was checked visually to ensure that the fracture had occurred through the femoral neck, the data was rejected ff the fracture had occurred outside the femoral neck. The load at fracture of the femoral neck was registered. Tibias were used for cortical bone testing after dissection and removal of the fibula just proximal to its insertion into the tibia. The tibia was then placed on two supports of an 8 mm span three point loading rig. The bone was positioned so the supports were under the curve in the proximal tibia and at the distal tibia so that when the load was applied at the mid point between the supports it was through the point of insertion of the fibula. Loading was applied at 0.1 mm s^−1^. In preliminary tests it was found that this position was stable, reproducible and that the tibia did not rotate during the test. The stiffness was measured over the linear portion of the loading curve and the load at failure was recorded.

### 5.9. Statistics

Morphological and micro-CT results are given as mean ± standard deviation (SD) and differences between CHAD−/− and WT animals were tested using a two-tailed independent Student's t-test. A multivariate analysis of variance (MANOVA) was used to compare immunogold data. For the latter, interest was focused on whether the overall distribution pattern for each of the two proteins differed between the groups. Thus, for a protein, only differences in overall comparison between the groups using MANOVA, and not difference in tests between subjects, were considered. A *p*-value of <0.05 was considered significant for all analyses.

## Supporting Information

Figure S1 **Immunostaining for COMP in the epiphyseal growth plate at 6** **weeks of age.** The epiphyseal growth plate (EGF) showed intense staining for COMP in both interterritorial and territorial matrix, although the staining did not differ by histologic scoring between CHAD−/− (3a) and wild type mice (3b). Non-immune control was negative (3c) (×10). Counterstained with H&E and phloxine B.
(TIF)

Figure S2 **Ultrastructural protein distribution of BSP in bone at 6** **weeks of age.** Sections incubated with anti-BSP showed distinct accumulation of gold particles over cement lines/mineralization fronts (arrows) in areas of mineralized bone but quantitative comparison revealed no differences in distribution pattern between the CHAD−/− (4a), wild type mice (4b) and non-immune control (4c) (TEM, ×49,000).
(TIF)

Figure S3 **Positions of the 3 sections analyzed in the femur of 4** **months old mice by micro-CT.**
(TIF)

Figure S4 **Threshold levels in comparison to the original grey scale scan for the 3 different age groups in the screening micro-CT experiment.**
(TIF)

Table S1 **The proteins identified by MASCOT 2.1 from 5457 peptide matches above homology or identity threshold, and ratios in CHAD−/− (KO) versus wild type (WT) mice.** Proteins identified by only one peptide were excluded. Proteins that changed ≥50% are underlined.
(DOCX)

Table S2 **Micro-CT cortical/trabecular bone parameters at different ages.** Bone length (femur), cortical/trabecular thickness (Ct.Th/Tb.Th), cortical volumetric BMD (Ct. vBMD), cortical/trabecular bone volume (Ct.BV/Tb.BV) cortical/trabecular porosity (Ct.P/Tb.P), trabecular separation (Tb.Sp), degree of anisotropy (DA) and structure model index (SMI) in proximal (P), middle (M) or distal (D) femur. ^*^ p<0.05, ^**^p<0.01 between CHAD−/− and wild type (WT) mice in the age-group.
(DOCX)

Table S3 **Oligonucleotide primer sequence for DIG-labeled cRNA probes.**
(DOCX)
