# Correction: The Skeletal Phenotype of Chondroadherin Deficient Mice

**DOI:** 10.1371/annotation/cb7d7b4c-624f-46e6-957e-13b355ca8e02

**Published:** 2013-07-02

**Authors:** Lovisa Hessle, Gunhild A. Stordalen, Christina Wenglén, Christiane Petzold, Elizabeth K. Tanner, Sverre-Henning Brorson, Espen S. Baekkevold, Patrik Önnerfjord, Finn P. Reinholt, Dick Heinegård

The name of the fifth author was given incorrectly. The correct name is: K. Elizabeth Tanner. The correct citation is: Hessle L, Stordalen GA, Wenglén C, Petzold C, Tanner KE, et al. (2013) The Skeletal Phenotype of Chondroadherin Deficient Mice. PLoS ONE 8(6): e63080. doi:10.1371/journal.pone.0063080. The correct abbreviation in Contributions is: KET. 

