# Supplementary figures and images for: The Skeletal Phenotype of Chondroadherin Deficient Mice
Source: PLoS One. 2013 Jun 3;8(6):e63080. doi: 10.1371/journal.pone.0063080 (PMC3670915; doi:10.1371/journal.pone.0063080)

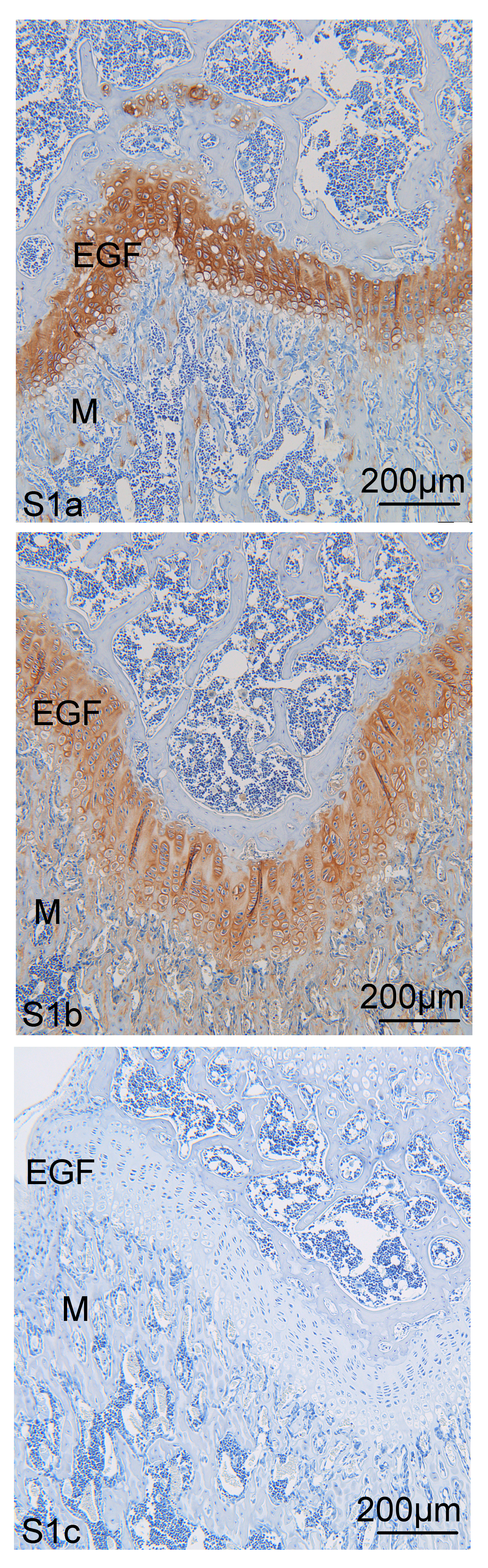

Supplement: Figure S1 — Immunostaining for COMP in the epiphyseal growth plate at 6 weeks of age. The epiphyseal growth plate (EGF) showed intense staining for COMP in both interterritorial and territorial matrix, although the staining did not differ by histologic scoring between CHAD−/− (3a) and wild type mice (3b). Non-immune control was negative (3c) (×10). Counterstained with H&E and phloxine B. (TIF) [file pone.0063080.s001.tif]

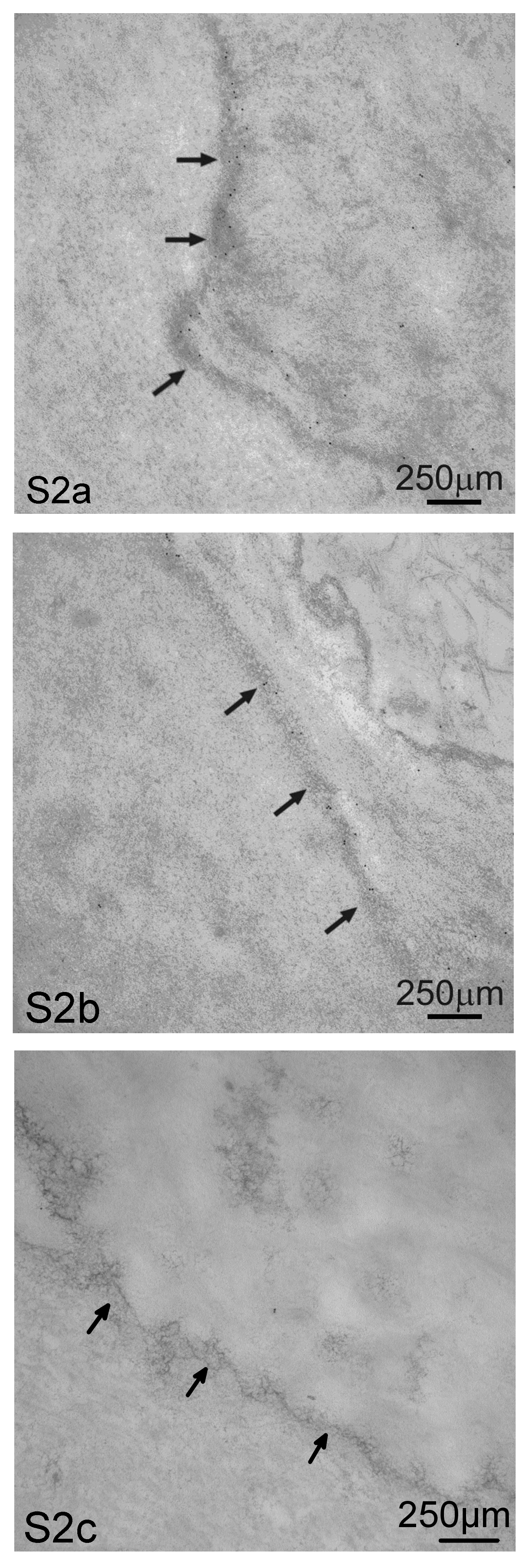

Supplement: Figure S2 — Ultrastructural protein distribution of BSP in bone at 6 weeks of age. Sections incubated with anti-BSP showed distinct accumulation of gold particles over cement lines/mineralization fronts (arrows) in areas of mineralized bone but quantitative comparison revealed no differences in distribution pattern between the CHAD−/− (4a), wild type mice (4b) and non-immune control (4c) (TEM, ×49,000). (TIF) [file pone.0063080.s002.tif]

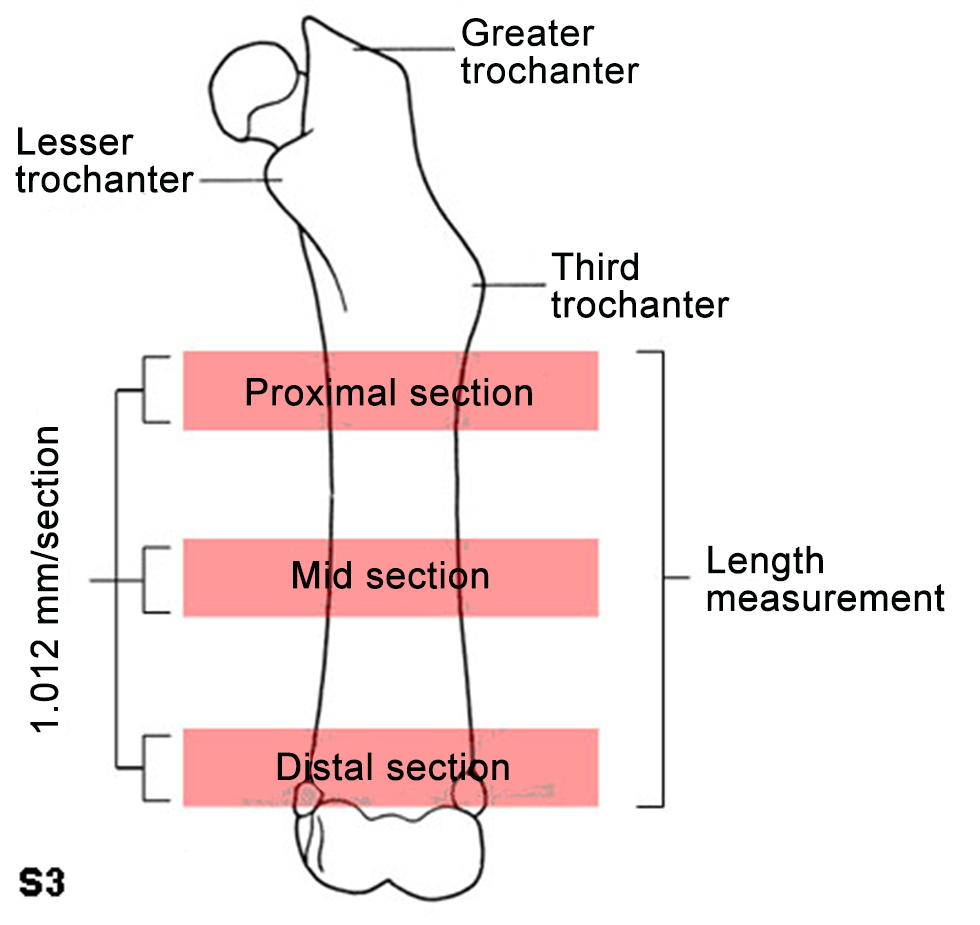

Supplement: Figure S3 — Positions of the 3 sections analyzed in the femur of 4 months old mice by micro-CT. (TIF) [file pone.0063080.s003.tif]

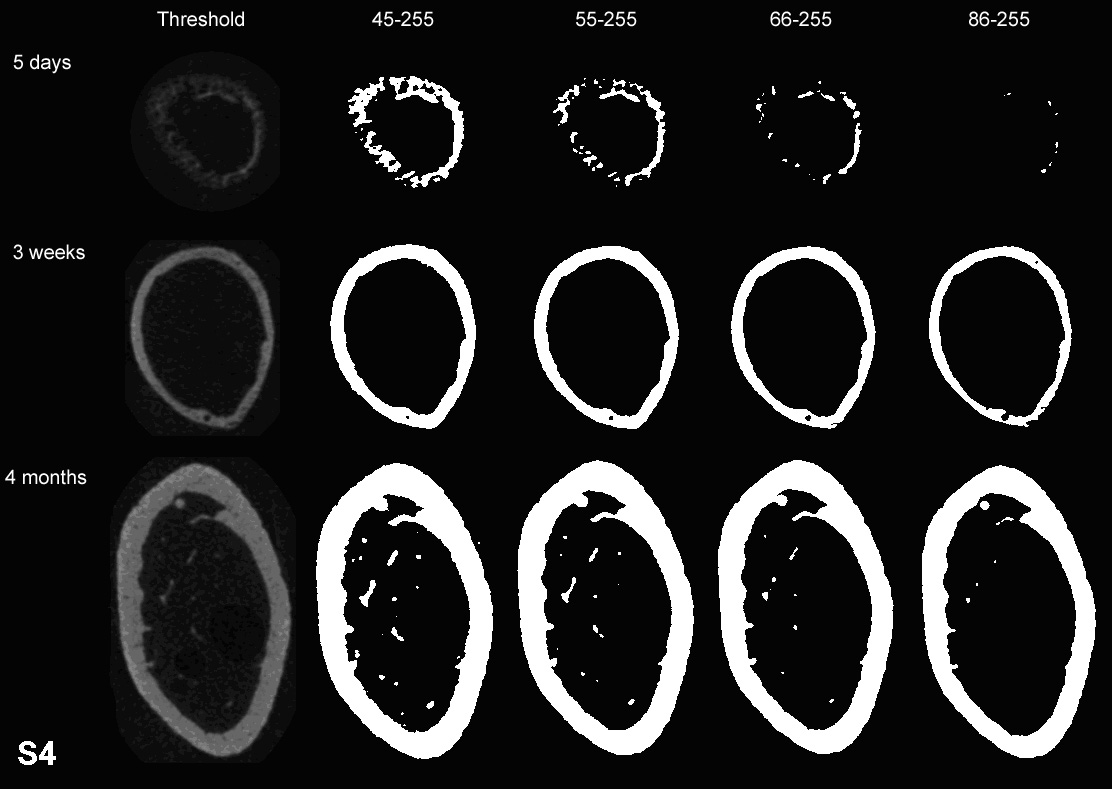

Supplement: Figure S4 — Threshold levels in comparison to the original grey scale scan for the 3 different age groups in the screening micro-CT experiment. (TIF) [file pone.0063080.s004.tif]
