# Supplementary material for: The Skeletal Phenotype of Chondroadherin Deficient Mice
Source: PLoS One. 2013 Jun 3;8(6):e63080. doi: 10.1371/journal.pone.0063080 (PMC3670915; doi:10.1371/journal.pone.0063080)
Supplement: Table S1 — The proteins identified by MASCOT 2.1 from 5457 peptide matches above homology or identity threshold, and ratios in CHAD−/− (KO) versus wild type (WT) mice. Proteins identified by only one peptide were excluded. Proteins that changed ≥50% are underlined. (DOCX) [file pone.0063080.s005.docx]

**Table S1 The proteins identified by MASCOT 2.1 from 5457 peptide matches above homology or identity threshold, and ratios in CHAD-/- (KO) versus wild type (WT) mice.** Proteins identified by only one peptide were excluded. Proteins that changed > 50 % are underlined

| **Acc. No.** | **Protein name** | **Female KO vs WT** | **Male KO vs WT** | **Female vs Male KO** | **Female vs Male WT** |
| --- | --- | --- | --- | --- | --- |
| P62259 | 14-3-3 protein epsilon | 0,68 | 0,33 | 1,72 | 0,83 |
| P63101 | 14-3-3 protein zeta/delta | 0,66 | 0,32 | 1,84 | 0,90 |
| P63325 | 40S ribosomal protein S10 | 0,97 | 0,73 | 0,88 | 0,66 |
| P62264 | 40S ribosomal protein S14 | 0,96 | 0,57 | 1,21 | 0,72 |
| P62270 | 40S ribosomal protein S18 | 0,99 | 0,71 | 0,97 | 0,70 |
| Q9CZX8 | 40S ribosomal protein S19 | 0,87 | 0,69 | 1,04 | 0,83 |
| P60867 | 40S ribosomal protein S20 | 0,90 | 0,66 | 0,84 | 0,61 |
| Q9CPR4 | 60S ribosomal protein L17 | 1,03 | 0,58 | 1,06 | 0,59 |
| P20029 | 78 kDa glucose-regulated protein | 0,94 | 0,69 | 1,23 | 0,90 |
| P68134 | Actin, alpha skeletal muscle | 1,01 | 0,62 | 1,74 | 1,07 |
| P60710 | Actin, cytoplasmic 1 | 1,01 | 0,60 | 1,79 | 1,07 |
| Q60604 | Adseverin | 0,64 | 0,52 | 1,33 | 1,08 |
| Q61282 | Aggrecan core protein | 1,00 | 0,82 | 1,07 | 0,88 |
| P09242 | Alkaline phosphatase, tissue-nonspecific isozyme | 0,93 | 0,79 | 1,02 | 0,86 |
| P07758 | Alpha-1-antitrypsin 1-1 | 0,37 | 0,25 | 1,41 | 0,95 |
| P22599 | Alpha-1-antitrypsin 1-2 | 0,39 | 0,27 | 1,37 | 0,94 |
| Q00896 | Alpha-1-antitrypsin 1-3 | 0,38 | 0,25 | 1,41 | 0,95 |
| Q00897 | Alpha-1-antitrypsin 1-4 | 0,38 | 0,27 | 1,35 | 0,96 |
| Q00898 | Alpha-1-antitrypsin 1-5 | 0,38 | 0,28 | 1,27 | 0,94 |
| P29699 | Alpha-2-HS-glycoprotein | 0,82 | 0,46 | 0,79 | 0,44 |
| P17182 | Alpha-enolase | 0,83 | 0,67 | 1,19 | 0,97 |
| P21570 | Angiogenin | 1,13 | 1,03 | 1,02 | 0,93 |
| P97429 | Annexin A4 | 0,82 | 0,77 | 1,09 | 1,02 |
| P48036 | Annexin A5 | 0,77 | 0,53 | 1,35 | 0,93 |
| P14824 | Annexin A6 | 0,84 | 0,72 | 1,16 | 0,99 |
| Q00623 | Apolipoprotein A-I | 1,20 | 0,62 | 1,14 | 0,59 |
| P08226 | Apolipoprotein E | 0,36 | 0,35 | 1,33 | 1,31 |
| Q03265 | ATP synthase subunit alpha, mitochondrial | 1,03 | 0,81 | 1,06 | 0,83 |
| P56480 | ATP synthase subunit beta, mitochondrial | 0,87 | 0,77 | 1,14 | 0,99 |
| P21550 | Beta-enolase | 0,99 | 0,85 | 1,19 | 1,02 |
| P28653 | Biglycan | 1,16 | 1,11 | 0,89 | 0,85 |
| P34821 | Bone morphogenetic protein 8A | 0,81 | 0,59 | 1,25 | 0,90 |
| P62204 | Calmodulin | 0,82 | 1,07 | 1,17 | 1,53 |
| O35887 | Calumenin | 0,88 | 0,86 | 1,03 | 1,00 |
| P16015 | Carbonic anhydrase 3 | 1,34 | 1,14 | 1,63 | 1,38 |
| Q66K08 | Cartilage intermediate layer protein 1 | 1,75 | 1,18 | 1,13 | 0,76 |
| P51942 | Cartilage matrix protein | 0,62 | 0,54 | 1,22 | 1,05 |
| Q9R0G6 | Cartilage oligomeric matrix protein | 0,99 | 0,89 | 1,01 | 0,91 |
| O55226 | Chondroadherin | 0,05 | 0,02 | 2,10 | 0,90 |
| Q9Z1F6 | Chondromodulin-1 | 0,97 | 0,49 | 1,52 | 0,76 |
| Q06890 | Clusterin | 0,90 | 0,91 | 1,01 | 1,02 |
| Q8BH61 | Coagulation factor XIII A chain | 0,70 | 0,60 | 1,09 | 0,93 |
| Q8R2G6 | Coiled-coil domain-containing protein 80 | 1,13 | 1,02 | 1,03 | 0,94 |
| P11087 | Collagen alpha-1(I) chain | 1,15 | 1,13 | 0,66 | 0,64 |
| P28481 | Collagen alpha-1(II) chain | 1,16 | 1,27 | 0,81 | 0,88 |
| P08121 | Collagen alpha-1(III) chain | 1,00 | 1,28 | 0,71 | 0,90 |
| Q05722 | Collagen alpha-1(IX) chain | 1,18 | 1,04 | 1,07 | 0,95 |
| Q04857 | Collagen alpha-1(VI) chain | 1,01 | 0,86 | 1,05 | 0,89 |
| Q05306 | Collagen alpha-1(X) chain | 1,05 | 0,94 | 0,91 | 0,81 |
| Q61245 | Collagen alpha-1(XI) chain | 1,01 | 1,24 | 0,73 | 0,90 |
| Q60847 | Collagen alpha-1(XII) chain | 1,35 | 1,13 | 1,19 | 1,00 |
| Q01149 | Collagen alpha-2(I) chain | 1,07 | 0,97 | 0,65 | 0,59 |
| Q07643 | Collagen alpha-2(IX) chain | 0,96 | 0,98 | 0,93 | 0,94 |
| Q3U962 | Collagen alpha-2(V) chain | 1,23 | 1,49 | 0,75 | 0,90 |
| Q02788 | Collagen alpha-2(VI) chain | 1,07 | 0,82 | 1,12 | 0,86 |
| Q64739 | Collagen alpha-2(XI) chain | 1,06 | 1,26 | 0,76 | 0,91 |
| P33435 | Collagenase 3 | 1,13 | 0,88 | 0,95 | 0,73 |
| Q04447 | Creatine kinase B-type | 0,96 | 1,13 | 0,99 | 1,17 |
| P07310 | Creatine kinase M-type | 0,92 | 1,20 | 0,97 | 1,27 |
| O88200 | C-type lectin domain family 11 member A | 0,59 | 0,71 | 1,10 | 1,32 |
| Q9EPW4 | C-type lectin domain family 3 member A | 1,34 | 1,02 | 1,01 | 0,76 |
| Q9WUQ5 | C-X-C motif chemokine 14 | 0,96 | 1,19 | 0,59 | 0,74 |
| P21460 | Cystatin-C | 0,82 | 0,64 | 0,93 | 0,72 |
| O35215 | D-dopachrome decarboxylase | 1,00 | 0,80 | 1,00 | 0,81 |
| P28654 | Decorin | 1,00 | 1,05 | 0,92 | 0,96 |
| P06802 | Ectonucleotide pyrophosphatase/phosphodiesterase family member 1 | 0,91 | 0,70 | 1,29 | 0,99 |
| P10126 | Elongation factor 1-alpha 1 | 0,78 | 0,65 | 1,01 | 0,84 |
| P70186 | Epiphycan | 0,84 | 0,90 | 0,93 | 1,00 |
| P63073 | Eukaryotic translation initiation factor 4E | 0,90 | 0,66 | 1,25 | 0,92 |
| Q8BGY2 | Eukaryotic translation initiation factor 5A-2 | 0,68 | 0,41 | 1,29 | 0,77 |
| P09528 | Ferritin heavy chain | 0,91 | 0,80 | 1,13 | 1,02 |
| P29391 | Ferritin light chain 1 | 1,05 | 0,97 | 1,05 | 0,97 |
| P50608 | Fibromodulin | 1,01 | 1,05 | 0,86 | 0,90 |
| P11276 | Fibronectin | 1,85 | 1,61 | 0,97 | 0,84 |
| P05064 | Fructose-bisphosphate aldolase A | 0,69 | 0,82 | 0,90 | 1,06 |
| P16110 | Galectin-3 | 0,83 | 0,78 | 1,06 | 0,99 |
| P13020 | Gelsolin | 0,57 | 0,54 | 1,26 | 1,19 |
| P46412 | Glutathione peroxidase 3 | 0,62 | 0,62 | 1,60 | 1,61 |
| P10649 | Glutathione S-transferase Mu 1 | 0,78 | 0,68 | 1,17 | 0,97 |
| P16858 | Glyceraldehyde-3-phosphate dehydrogenase | 0,79 | 0,66 | 1,15 | 0,95 |
| P63017 | Heat shock cognate 71 kDa protein | 1,00 | 0,72 | 1,18 | 0,85 |
| P01942 | Hemoglobin subunit alpha | 0,69 | 0,55 | 0,92 | 0,74 |
| P02088 | Hemoglobin subunit beta-1 | 0,62 | 0,41 | 1,12 | 0,74 |
| Q91X72 | Hemopexin | 1,25 | 0,70 | 0,73 | 0,41 |
| Q99020 | Heterogeneous nuclear ribonucleoprotein A/B | 0,75 | 0,72 | 0,76 | 0,73 |
| Q8BG05 | Heterogeneous nuclear ribonucleoprotein A3 | 0,79 | 0,61 | 0,79 | 0,67 |
| Q9Z130 | Heterogeneous nuclear ribonucleoprotein D-like | 0,74 | 0,75 | 0,78 | 0,79 |
| O88569 | Heterogeneous nuclear ribonucleoproteins A2/B1 | 0,81 | 0,73 | 0,80 | 0,73 |
| P10922 | Histone H1.0 | 0,85 | 0,86 | 0,98 | 0,99 |
| P43275 | Histone H1.1 | 0,99 | 0,85 | 0,97 | 0,83 |
| P15864 | Histone H1.2 | 0,98 | 0,87 | 0,96 | 0,86 |
| P43277 | Histone H1.3 | 0,99 | 0,88 | 0,95 | 0,84 |
| P43274 | Histone H1.4 | 0,99 | 0,88 | 0,95 | 0,84 |
| P43276 | Histone H1.5 | 1,02 | 0,88 | 0,93 | 0,79 |
| Q8BFU2 | Histone H2A type 3 | 0,96 | 0,86 | 0,92 | 0,82 |
| P27661 | Histone H2A.x | 0,96 | 0,87 | 0,91 | 0,82 |
| P10853 | Histone H2B type 1-F/J/L | 0,98 | 0,83 | 0,97 | 0,82 |
| P10854 | Histone H2B type 1-M | 0,98 | 0,83 | 0,97 | 0,82 |
| P68433 | Histone H3.1 | 1,09 | 0,73 | 1,07 | 0,72 |
| P62806 | Histone H4 | 1,22 | 0,96 | 1,23 | 0,97 |
| Q9QUP5 | Hyaluronan and proteoglycan link protein 1 | 0,88 | 0,85 | 0,99 | 0,95 |
| Q9D819 | Inorganic pyrophosphatase | 0,64 | 0,58 | 0,90 | 0,71 |
| Q6P9L6 | Kinesin-like protein KIF15 | 1,15 | 0,95 | 1,15 | 0,95 |
| P21956 | Lactadherin | 1,32 | 1,26 | 0,97 | 0,92 |
| P48678 | Lamin-A/C | 1,01 | 0,85 | 1,14 | 0,96 |
| P06151 | L-lactate dehydrogenase A chain | 0,51 | 0,35 | 1,42 | 0,99 |
| P51885 | Lumican | 0,98 | 0,92 | 0,98 | 0,92 |
| Q924C6 | LysylOxidase homolog 4 | 1,00 | 0,90 | 1,09 | 0,99 |
| P14152 | Malate dehydrogenase, cytoplasmic | 0,77 | 0,53 | 1,27 | 0,87 |
| O35701 | Matrilin-3 | 0,88 | 1,04 | 0,94 | 1,11 |
| P19788 | Matrix Gla protein | 1,07 | 0,99 | 1,23 | 1,15 |
| Q62000 | Mimecan | 0,80 | 0,89 | 0,83 | 0,94 |
| Q6PCP5 | Mitochondrial fission factor | 1,04 | 0,80 | 1,04 | 0,81 |
| P26041 | Moesin | 0,86 | 0,61 | 1,24 | 0,88 |
| O70624 | Myocilin | 0,78 | 0,56 | 0,94 | 0,68 |
| P04247 | Myoglobin | 1,00 | 1,15 | 1,16 | 1,33 |
| P05977 | Myosin light chain 1, skeletal muscle isoform | 0,90 | 1,29 | 1,04 | 1,49 |
| P97457 | Myosin regulatory light chain 2, skeletal muscle isoform | 0,95 | 1,35 | 1,06 | 1,51 |
| Q5SX40 | Myosin-1 | 1,00 | 1,19 | 1,16 | 1,38 |
| Q5SX39 | Myosin-4 | 0,94 | 1,19 | 1,11 | 1,41 |
| P13542 | Myosin-8 | 1,03 | 1,23 | 1,12 | 1,34 |
| O88322 | Nidogen-2 | 0,97 | 0,88 | 0,93 | 0,84 |
| Q02819 | Nucleobindin-1 | 0,90 | 0,68 | 1,06 | 0,79 |
| P81117 | Nucleobindin-2 | 0,85 | 0,76 | 1,02 | 0,91 |
| Q8R480 | Nucleoporin NUP85 | 0,77 | 0,63 | 1,20 | 0,99 |
| Q01768 | Nucleoside diphosphate kinase B | 0,85 | 0,59 | 1,24 | 0,84 |
| O35103 | Osteomodulin | 0,77 | 1,02 | 0,80 | 1,06 |
| P17742 | Peptidyl-prolyl cis-trans isomerase A | 0,95 | 0,74 | 1,11 | 0,86 |
| P24369 | Peptidyl-prolyl cis-trans isomerase B | 0,92 | 0,80 | 1,03 | 0,89 |
| Q05793 | Perlecan | 0,93 | 0,82 | 1,00 | 0,88 |
| P35700 | Peroxiredoxin-1 | 0,68 | 0,51 | 1,24 | 0,94 |
| P99029 | Peroxiredoxin-5, mitochondrial | 0,73 | 0,82 | 0,77 | 0,87 |
| O08709 | Peroxiredoxin-6 | 0,81 | 0,71 | 1,21 | 1,02 |
| P70296 | Phosphatidylethanolamine-binding protein 1 | 0,65 | 0,67 | 1,05 | 1,08 |
| P09411 | Phosphoglycerate kinase 1 | 0,75 | 0,65 | 1,14 | 0,98 |
| Q9DBJ1 | Phosphoglycerate mutase 1 | 0,78 | 0,56 | 1,30 | 0,93 |
| P22777 | Plasminogen activator inhibitor 1 | 0,90 | 0,58 | 1,45 | 0,93 |
| Q9JK53 | Prolargin | 1,10 | 1,17 | 0,94 | 1,00 |
| Q60715 | Prolyl 4-hydroxylase subunit alpha-1 | 0,84 | 0,70 | 1,11 | 0,92 |
| Q60716 | Prolyl 4-hydroxylase subunit alpha-2 | 0,76 | 0,61 | 1,23 | 0,99 |
| P09103 | Protein disulfide-isomerase | 0,72 | 0,58 | 1,34 | 1,09 |
| P27773 | Protein disulfide-isomerase A3 | 0,78 | 0,70 | 1,11 | 1,01 |
| Q922R8 | Protein disulfide-isomerase A6 | 0,64 | 0,61 | 1,10 | 1,06 |
| P31725 | Protein S100-A9 | 1,02 | 0,21 | 1,08 | 0,22 |
| Q9JM99 | Proteoglycan 4 | 1,16 | 0,96 | 1,03 | 0,86 |
| P52480 | Pyruvate kinase isozymes M1/M2 | 0,72 | 0,62 | 1,09 | 0,94 |
| P26043 | Radixin | 0,85 | 0,60 | 1,22 | 0,87 |
| Q05186 | Reticulocalbin-1 | 0,76 | 0,74 | 1,00 | 0,97 |
| Q8BH97 | Reticulocalbin-3 | 1,00 | 0,73 | 1,27 | 0,92 |
| Q00724 | Retinol-binding protein 4 | 0,92 | 0,65 | 1,30 | 0,91 |
| Q99PT1 | Rho GDP-dissociation inhibitor 1 | 0,82 | 0,75 | 1,09 | 1,01 |
| Q9JJH1 | Ribonuclease 4 | 1,30 | 0,92 | 1,02 | 0,72 |
| P07759 | Serine protease inhibitor A3K | 0,94 | 0,70 | 0,47 | 0,35 |
| Q921I1 | Serotransferrin | 1,00 | 0,76 | 1,04 | 0,79 |
| P19324 | Serpin H1 | 0,84 | 0,62 | 1,12 | 0,83 |
| P07724 | Serum albumin | 1,29 | 0,81 | 1,13 | 0,71 |
| Q9CYR0 | Single-stranded DNA-binding protein, mitochondrial | 0,58 | 0,57 | 1,25 | 1,19 |
| P62320 | Small nuclear ribonucleoprotein Sm D3 | 0,89 | 0,58 | 0,96 | 0,63 |
| P07214 | SPARC | 1,03 | 0,93 | 0,79 | 0,71 |
| Q8CD91 | SPARC-related modular calcium-binding protein 2 | 1,08 | 1,19 | 0,84 | 0,92 |
| P08228 | Superoxide dismutase [Cu-Zn] | 0,92 | 0,86 | 0,98 | 0,91 |
| Q8R054 | Sushi repeat-containing protein SRPX2 | 0,94 | 0,97 | 0,84 | 0,87 |
| O08599 | Syntaxin-binding protein 1 | 0,92 | 0,78 | 1,05 | 0,89 |
| P80315 | T-complex protein 1 subunit delta | 0,43 | 0,42 | 1,27 | 1,23 |
| Q91W90 | Thioredoxin domain-containing protein 5 | 0,74 | 0,59 | 1,05 | 0,91 |
| P35441 | Thrombospondin-1 | 0,69 | 0,74 | 1,01 | 1,08 |
| Q9Z1T2 | Thrombospondin-4 | 1,09 | 0,87 | 1,17 | 0,93 |
| P40142 | Transketolase | 1,12 | 0,74 | 1,26 | 0,84 |
| P63028 | Translationally-controlled tumor protein | 0,99 | 0,77 | 1,02 | 0,80 |
| P17751 | Triosephosphate isomerase | 0,80 | 0,66 | 1,13 | 0,93 |
| P58771 | Tropomyosin alpha-1 chain | 1,09 | 1,57 | 1,21 | 1,73 |
| P58774 | Tropomyosin beta chain | 1,18 | 1,79 | 1,29 | 1,91 |
| P13412 | Troponin I, fast skeletal muscle | 1,04 | 1,61 | 1,10 | 1,71 |
| Q9QZ47 | Troponin T, fast skeletal muscle | 0,90 | 1,74 | 0,98 | 1,93 |
| Q7TMM9 | Tubulin beta-2A chain | 0,89 | 0,31 | 1,64 | 0,58 |
| P62991 | Ubiquitin | 0,91 | 0,86 | 0,96 | 0,90 |
| P20152 | Vimentin | 0,77 | 0,73 | 0,95 | 0,90 |
| Q8VHI5 | Vitrin | 0,75 | 1,18 | 0,84 | 1,32 |
