# Supplementary material for: The Skeletal Phenotype of Chondroadherin Deficient Mice
Source: PLoS One. 2013 Jun 3;8(6):e63080. doi: 10.1371/journal.pone.0063080 (PMC3670915; doi:10.1371/journal.pone.0063080)
Supplement: Table S2 — Micro-CT cortical/trabecular bone parameters at different ages. Bone length (femur), cortical/trabecular thickness (Ct.Th/Tb.Th), cortical volumetric BMD (Ct. vBMD), cortical/trabecular bone volume (Ct.BV/Tb.BV) cortical/trabecular porosity (Ct.P/Tb.P), trabecular separation (Tb.Sp), degree of anisotropy (DA) and structure model index (SMI) in proximal (P), middle (M) or distal (D) femur. * p<0.05, **p<0.01 between CHAD−/− and wild type (WT) mice in the age-group. (DOCX) [file pone.0063080.s006.docx]

**Table S2 Micro-CT cortical/trabecular bone parameters at different ages**

|  | 5 days | | 3 weeks | | 4 months | |
| --- | --- | --- | --- | --- | --- | --- |
| Parameter | CHAD-/- | WT | CHAD-/- | WT | CHAD-/- | WT |
| Bone length (mm) | 4.55 ± 0.02 | 4.33 ± 0.43 | 9.01 ± 0.29 | 9.30 ± 0.29 | 14.59 ± 0.71 | 14.88 ± 0.46 |
| Ct.Th (mm) (P) | 0.04 ± 0.00 | 0.04 ± 0.01 | 0.07 ± 0.01 | 0.06 ± 0.00 | 0.17 ± 0.00^**^ | 0.18 ± 0.00 |
| Ct.vBMD (g/cm^-3^) (M) | 0.42 ± 0.01 | 0.55 ± 0.14 | 1.35 ± 0.09 | 1.34 ± 0.05 | 1.53 ± 0.05^*^ | 1.38 ± 0.04 |
| Ct.BV (%) (M) | 59.67 ± 3.42 | 78.23 ± 17.67 | 98.89 ± 0.13^**^ | 98.18 ± 0.17 | 99.61 ± 0.02 | 99.14 ± 0.54 |
| Ct.P (%) (M) | 40.33 ± 3.42 | 21.77 ± 17.67 | 1.11 ± 0.13^**^ | 1.82 ± 0.17 | 0.39 ± 0.02 | 0.86 ± 0.54 |
| Tb.BV (%) (D) | 12.40 ± 3.43 | 16.94 ± 7.00 | 2.44 ± 0.28 | 2.67 ± 1.07 | 3.57 ± 1.74 | 2.67 ± 0.82 |
| Tb.P (%) (D) | 87.60 ± 3.43 | 83.06 ± 7.00 | 97.56 ± 0.28 | 97.33 ± 1.07 | 95.19 ± 2.34 | 96.40 ± 1.11 |
| Tb.Th (mm) (D) | 0.036 ± 0.00 | 0.039 ± 0.00 | 0.037 ± 0.00 | 0.036 ± 0.00 | 0.046 ± 0.01 | 0.050 ± 0.00 |
| Tb.Sp (mm) (D) | 0.155 ± 0.01 | 0.157 ± 0.03 | 0.349 ± 0.01 | 0.343 ± 0.05 | 0.350 ± 0.10 | 0.407 ± 0.04 |
| DA (D) | 2.03 ± 0.38 | 2.42 ± 0.28 | 2.23 ± 0.67 | 2.84 ± 0.43 | 98.78 ± 12.31 | 95.53 ± 24.7 |
| SMI (D) | 2.38 ± 0.32 | 2.02 ± 0.39 | 2.69 ± 0.17 | 2.57 ± 0.12 | 2.36 ± 0.18 | 2.24 ± 0.56 |

Bone length (femur), cortical/trabecular thickness (Ct.Th/Tb.Th), cortical volumetric BMD (Ct. vBMD), cortical/trabecular bone volume (Ct.BV/Tb.BV) cortical/trabecular porosity (Ct.P/Tb.P), trabecular separation (Tb.Sp), degree of anisotropy (DA) and structure model index (SMI) in proximal (P), middle (M) or distal (D) femur. ^*^ p<0.05, ^**^p<0.01 between CHAD-/- and wild type (WT) mice in the age-group.
