# Supplementary material for: The Skeletal Phenotype of Chondroadherin Deficient Mice
Source: PLoS One. 2013 Jun 3;8(6):e63080. doi: 10.1371/journal.pone.0063080 (PMC3670915; doi:10.1371/journal.pone.0063080)
Supplement: Table S3 — Oligonucleotide primer sequence for DIG-labeled cRNA probes. (DOCX) [file pone.0063080.s007.docx]

**Table S3 Oligonucleotide primer sequence for DIG-labeled cRNA probes**

| **Sequence name** | **Sequence forward** | **Sequence reverse** |
| --- | --- | --- |
| **COMP** | 5’-taacccagaccagagggatg-3’ | 5’-ccgttgaaggctgtgtaacc-3’ |
| **TRAP** | 5’-acttgcgaccattgttagcc-3’ | 5’-ctgtgggatcagttggtgtg-3’ |
| **CTK** | 5’-ccagtgggagctatggaaga-3’ | 5-tctgctgcacgtattggaag-3’ |
| **OPN** | 5’-cgatgatgatgacgatggag-3’ | 5’-ttgtggctctgatgttccag-3’ |
| **BSP** | 5’-gaagcaggtgcagaaggaac-3’ | 5’-agcatttgcggaaatcactc-3’ |
